# Distinct influenza interfaces in cattle: repeated spillovers of highly pathogenic avian influenza A(H5N1) from wild birds and enzootic circulation of influenza D virus

**DOI:** 10.3389/fcimb.2026.1957795

**Published:** 2026-09-22

**Authors:** Siqi Wang, Kaili Gao, Lianmin Li, Yiming Chang, Luyu Mao, Shuchang Cheng, Yang Bai, Shuya Yang, Sainan Li, Yongli Guo, Mingchun Gao

**Affiliations:** 1 College of Veterinary Medicine, Northeast Agricultural University, Harbin, Heilongjiang, China; 2 Department of Immunology, School of Basic Medical Sciences, Harbin Medical University, Harbin, Heilongjiang, China

**Keywords:** bovine respiratory disease complex, cattle, cross-species transmission, highly pathogenic avian influenza a(H5N1), influenza D virus, mammary tropism, one health surveillance

## Abstract

Cattle now occupy two epidemiologically distinct influenza interfaces. This Mini Review comparatively synthesizes epidemiological, pathological, experimental-transmission, genomic, and surveillance evidence for highly pathogenic avian influenza A(H5N1) and influenza D virus (IDV), asking how source ecology, tissue tropism, shedding, transmission, persistence, and zoonotic evidence differ between the two viruses. Genetically distinct H5N1 viruses have spilled over repeatedly from wild birds into United States dairy cattle. In lactating cows, infection is prominent in mammary alveolar epithelium and produces high titers of infectious virus in raw milk; inter-herd cattle movements have disseminated genotype B3.13 through dairy production networks. On affected farms, viral RNA has been detected in milk and respiratory specimens; infectious virus is recovered consistently from raw milk and mammary tissues but only sporadically from respiratory or environmental samples such as milking-parlour air and wastewater. These findings identify exposure settings but do not resolve the dominant natural cow-to-cow transmission route or how the virus reaches the mammary gland. Occupational infections have occurred, but sustained cattle adaptation or long-term reservoir status has not been demonstrated. In contrast, IDV is a cattle-adapted respiratory virus maintained through direct contact and short-range airborne transmission, with interlineage reassortment among IDV genome segments and frequent association with bovine respiratory disease complex (BRDC), although an independent causal role in BRDC remains unproven. Human seroreactivity and efficient replication in human airway models indicate exposure and biological compatibility, but not confirmed productive human infection or onward transmission. These contrasts support event-triggered, milk-centered investigation for H5N1 and longitudinal respiratory and genomic surveillance for IDV, built on shared One Health diagnostic, sequencing, epidemiological, and occupational-health infrastructure.

## 1 Introduction

Influenza A virus ecology is commonly framed around wild aquatic birds as the principal natural reservoir, with poultry, swine, and humans occupying different host roles in amplification, reassortment, adaptation, and disease emergence (Krammer et al., 2018). Cattle received little attention because sustained influenza A virus circulation had not been documented in bovine populations. In 2024, clade 2.3.4.4b highly pathogenic avian influenza A(H5N1) [HPAI A(H5N1)] was confirmed in United States dairy cattle (Caserta et al., 2024), and genomic analysis traced spread through interconnected dairy production networks (Nguyen et al., 2025). A joint assessment by the Food and Agriculture Organization of the United Nations (FAO), World Health Organization (WHO), and World Organization for Animal Health (WOAH), based on data available through 1 March 2026, documented several independent spillovers from wild birds into cattle, demonstrating that multiple viral genotypes can infect this host (Food and Agriculture Organization of the United Nations et al., 2026).

The emergence of H5N1 in dairy cattle established a production-linked influenza interface centered on lactation, milking, and cattle movement. Field and experimental studies documented mammary infection, high virus titers in milk, mastitis, abnormal milk, and production loss (Caserta et al., 2024; Baker et al., 2025; Peña-Mosca et al., 2025). Movement of infected cattle facilitated long-distance dissemination (Nguyen et al., 2025), whereas genetically distinct spillovers from wild birds created new points of entry (Food and Agriculture Organization of the United Nations et al., 2026). Thus, raw milk and milking procedures are central exposure settings, cattle movement links herds, and contaminated equipment, aerosols, wastewater, cross-species contact, and occupational contact define additional exposure pathways whose contributions to natural transmission remain unresolved.

Influenza D virus (IDV) follows a different ecological pattern. First isolated from a pig with respiratory disease (Hause et al., 2013), it was later established as a distinct orthomyxovirus with cattle as its principal maintenance host (Hause et al., 2014). Experimental studies in calves demonstrated direct-contact (Ferguson et al., 2016) and short-range airborne transmission (Salem et al., 2019). Case-control (Ng et al., 2015) and cohort (Nissly et al., 2020) studies associate IDV with bovine respiratory disease complex (BRDC) without establishing independent or dominant causation. Whole-genome studies document diversification and geographic structure (Limaye et al., 2024), interlineage reassortment among IDV genome segments (Collin et al., 2015), and regional persistence (Li H. et al., 2026), favoring longitudinal surveillance over outbreak-limited investigation.

The two viruses differ most clearly in their principal replication sites. Mammary replication links H5N1 to milk and dairy production networks, whereas respiratory replication links IDV to enzootic circulation and BRDC. We therefore compare shedding, transmission, disease context, evolutionary dynamics, and surveillance across these compartments, while distinguishing occupational exposure signals from evidence of productive human infection or onward transmission.

## 2 Highly pathogenic avian influenza A(H5N1) in dairy cattle: emergence, mammary infection, and unresolved transmission routes

The United States cattle epizootic was initially dominated by genotype B3.13. Phylogenetic and epidemiological analysis support a single wild-bird-to-bovine spillover followed by extensive cattle-associated, movement-linked spread (Nguyen et al., 2025). Genotype D1.1 detections in Nevada and Arizona represented two additional independent spillovers from wild birds, each with limited cattle transmission (Food and Agriculture Organization of the United Nations et al., 2026). Repeated entry and sustained circulation are therefore separate processes. Unresolved questions are how often wild-bird-derived H5N1 crosses into cattle, which changes arise after spillover, and which routes sustain transmission. Extensive cattle-associated spread alone does not establish long-term reservoir status.

After introduction, the distinctive pathological feature in lactating cows is mammary infection. Field investigations detected abundant viral RNA and infectious virus in milk, epithelial necrosis, and H5N1 antigen by immunohistochemistry in mammary alveolar epithelial cells (Caserta et al., 2024). Intramammary inoculation reproduced severe mastitis, abnormal milk, reduced production, and high-titer milk shedding (Baker et al., 2025). In contrast, oronasal inoculation of calves produced moderate upper-respiratory replication and nasal shedding, without detected transmission to sentinels (Halwe et al., 2025). Because these experiments differed in age, lactation status, target tissue, and inoculation route, they establish mammary tropism but do not identify the natural entry route.

Transmission evidence remains less definitive. Low-dose intramammary challenge caused robust infection and high-titer shedding in milk, but transmission to sentinel cows was not detected despite repeated exposure to contaminated milking equipment and close contact. Respiratory and oral exposures were also less efficient than intramammary inoculation at establishing productive infection under the tested conditions (Lee et al., 2026). Across 14 California dairy farms, infectious virus was recovered from milking-parlour air on three farms and from wastewater on one farm; viral RNA was also detected in exhaled breath and milk from cows without overt clinical signs (Campbell et al., 2026). The persistent, spatially heterogeneous distribution of positive udder quarters suggested that shared milking equipment was unlikely to be the sole route. Collectively, these observations identify plausible exposure settings but do not resolve the natural portal of entry or the route by which the virus reaches the mammary gland.

Mechanistic studies have examined why mammary tissue is permissive. Plant lectins and haemagglutinin probes identified glycans compatible with contemporary avian H5 binding, but gave discordant signals for putative human-type receptors; lectin staining cannot establish functional receptor use (Kristensen et al., 2024; Rios Carrasco et al., 2024). Glycan binding also cannot explain route-specific infection or farm transmission. A bovine clade 2.3.4.4b isolate replicated in mammary and airway organoids without demonstrating respiratory transmission *in vivo* (Li C. et al., 2026). Intramammary challenge elicited compartmentalized cytokine responses, systemic myeloid remodeling, lymphocyte activation, and increased H5- and N1-specific antibody titers (Singh et al., 2026). Protease availability, local innate immunity, tissue physiology, dose, and lactation stage may also contribute.

The consequences of mammary infection extend from production loss to cross-species exposure. In one affected herd, about one fifth of adult cows developed clinical disease, and average milk production per affected cow was estimated to be 901 kg lower than expected during the 60 days after diagnosis (Peña-Mosca et al., 2025). Because this estimate comes from a single herd, it should not be extrapolated across management systems. Raw milk or colostrum from infected cows can also expose other species: farm cats fed these products developed fatal systemic and neurological disease, and closely related viruses were found in cattle and cats on the same premises (Burrough et al., 2024).

For people, the clearest evidence is occupational spillover. The first recognized infection involved a Texas dairy worker who developed conjunctivitis after exposure to affected cattle (Uyeki et al., 2024). Additional infections were later identified among dairy workers, who usually presented with conjunctivitis or mild respiratory illness (Garg et al., 2025). These cases do not demonstrate sustained human-to-human transmission. The May 2026 FAO/WHO/WOAH assessment rated the global risk to the general population as low and the risk to occupationally or frequently exposed people as low to moderate, depending on local epidemiology and protective measures (Food and Agriculture Organization of the United Nations et al., 2026). A WHO update covering reports available through 7 July 2026 likewise found no sustained human-to-human transmission of A(H5) viruses (World Health Organization, 2026). Validated commercial pasteurization conditions effectively inactivate H5N1, whereas raw milk from infected cattle may expose people and animals to infectious virus (United States Food and Drug Administration, 2026).

At the viral level, replication in cattle creates a new selective environment for H5N1. Early in the outbreak, polymerase substitutions including PB2 M631L and PA K497R became enriched among cattle-associated viruses and were shown to increase polymerase activity in bovine cells and enhance viral replication in mammary tissue models (Dholakia et al., 2026). However, these substitutions have not been shown to confer efficient bovine respiratory or airborne transmission, and their detection alone is insufficient to infer pandemic potential (Dholakia et al., 2026; Food and Agriculture Organization of the United Nations et al., 2026). Substitutions that recur or increase in frequency should be evaluated phenotypically in relevant bovine and other mammalian models.

## 3 Influenza D virus in cattle: enzootic respiratory circulation, BRDC, and evolution

IDV was first isolated in 2011 from a pig with respiratory disease and described as influenza C-like (Hause et al., 2013). Subsequent work established IDV as a distinct orthomyxovirus and identified cattle as its principal maintenance host, based partly on substantially higher seroprevalence in cattle than in swine (Hause et al., 2014). Surveillance has since documented broad but uneven circulation in bovine populations (Collin et al., 2015; Limaye et al., 2024; Li H. et al., 2026).

In experimentally infected cattle, IDV replicates in the upper and lower airways, causes mild clinical signs and tracheal lesions, and transmits to direct-contact animals (Ferguson et al., 2016). In another experiment, sentinel calves housed 3 m from aerosol-inoculated animals became infected despite the absence of direct or fomite contact, supporting short-range airborne transmission (Salem et al., 2019). Together, these experiments support direct-contact and short-range airborne transmission. Transport and commingling may amplify transmission under field conditions, although their contributions to the long-term maintenance of IDV in cattle populations have not been quantified.

IDV is transmissible among cattle, but its independent role in BRDC remains uncertain. A metagenomic case-control study detected IDV more often in cattle with severe respiratory disease than in matched controls (Ng et al., 2015). A later cohort linked higher IDV loads and a greater burden of viral coinfection with clinical disease (Nissly et al., 2020). BRDC is nevertheless multifactorial and is shaped by transport stress, crowding, host immune status, bacterial colonization, management practices, and multiple viral infections. An IDV-positive RT-qPCR result confirms the presence of viral RNA in the tested respiratory specimen but does not by itself distinguish active infection from transient carriage or establish that IDV caused the clinical syndrome.

Coinfection experiments provide evidence of disease enhancement. Calves infected with both IDV and *Mycoplasma bovis* developed earlier and more severe disease than calves given either agent alone, and IDV promoted *M. bovis* colonization of the lower respiratory tract (Lion et al., 2021). These findings support a contributory role for IDV within the BRDC pathogen network, although the magnitude of that contribution remains uncertain. Prospective studies should relate viral kinetics to airway lesions, bacterial colonization of the lower respiratory tract, host responses, and clinical outcomes.

Enzootic circulation provides repeated opportunities for diversification. Early work identified cocirculating D/OK and D/660 lineages and interlineage reassortment among the seven IDV genome segments (Collin et al., 2015). Subsequent whole-genome analysis revealed further diversification, geographic structure, and additional interlineage reassortment (Limaye et al., 2024). In Northeast China, D/Yamagata/2019-lineage viruses were detected only sporadically, yet phylogenetic continuity indicated ongoing circulation (Li H. et al., 2026). Because reassortment can involve any of the seven segments, tracing viral movement and segment exchange requires whole-genome sequencing rather than analysis of the haemagglutinin-esterase-fusion gene alone (Collin et al., 2015; Limaye et al., 2024).

Several studies indicate human exposure, but clinical significance is uncertain. Neutralizing antibodies were more frequent in cattle-exposed adults than controls (White et al., 2016). A cross-shift study detected IDV RNA in dairy-worker nasal washes without association with symptoms and could not distinguish contamination, carriage, and productive infection (Leibler et al., 2023). IDV replicates in differentiated human airway epithelium, showing cellular permissiveness without disease (Holwerda et al., 2019). A bovine isolate transmitted efficiently between ferrets; the same study reported unusually high seroprevalence in Northeast China that requires independent validation (Gao et al., 2025). Another study found that bovine and swine isolates replicated efficiently in lung epithelial cell lines, differentiated airway cultures, and precision-cut human lung slices while eliciting limited innate responses (Sanders et al., 2026). These experimental findings support mammalian transmission and human-airway compatibility but not naturally acquired human disease or onward transmission. No IDV-attributable human disease burden has been defined.

## 4 Comparing the two influenza interfaces in cattle

H5N1 and IDV occupy distinct ecological roles in cattle, defined less by their shared host than by their principal sites of replication. H5N1 enters cattle through spillover from wild birds; prominent mammary infection results in high-titer viral shedding in milk, while animal movements facilitate subsequent dissemination. IDV is maintained by respiratory transmission, diversifies during enzootic circulation, and interacts with the polymicrobial pathogen network of BRDC. The comparison is ecological and does not imply taxonomic or pathogenic equivalence (**Figure 1**).

**Figure 1:**
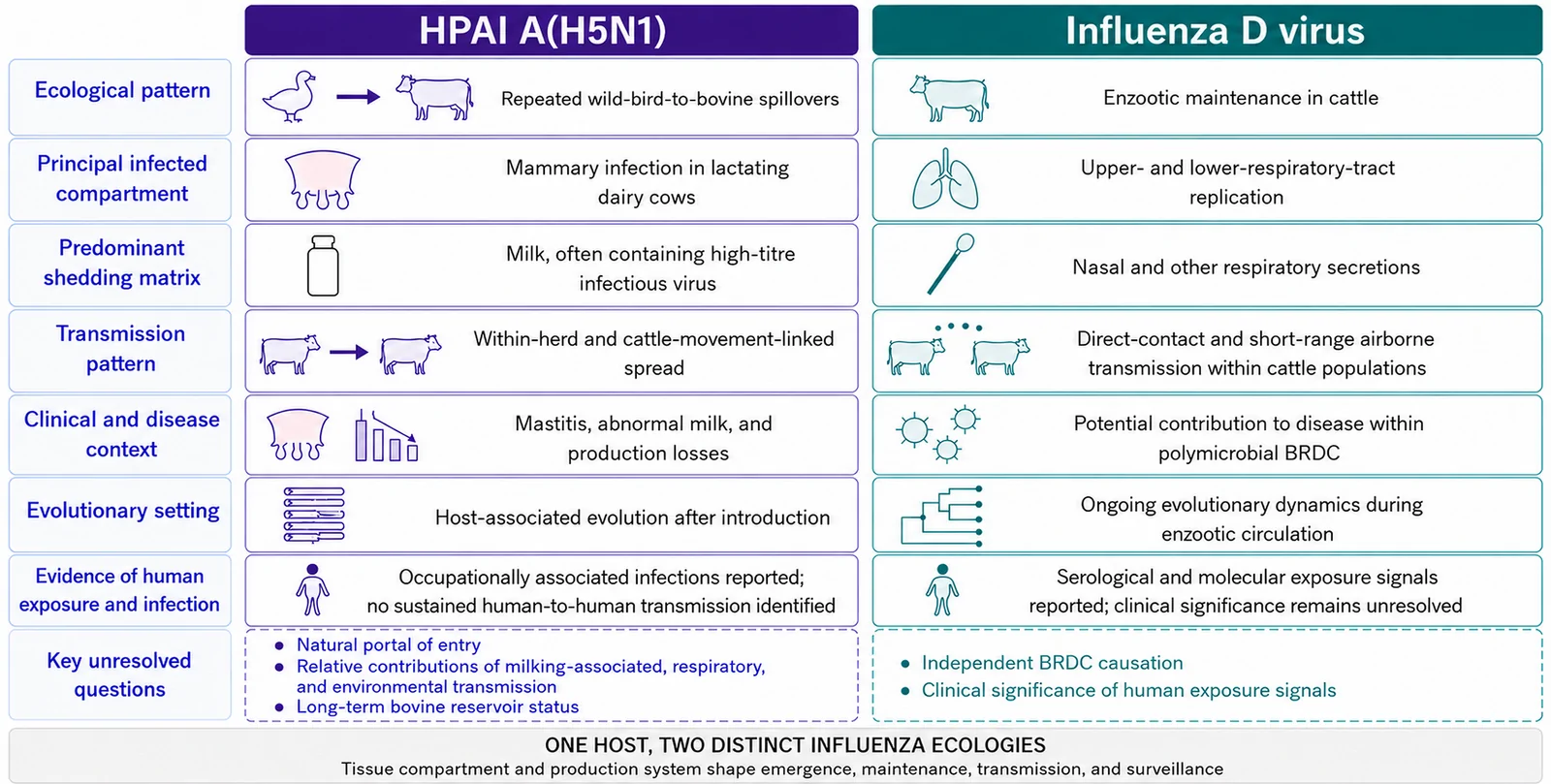
Two influenza interfaces in cattle. Repeated spillovers of HPAI A(H5N1) from wild birds have introduced the virus into dairy cattle populations. Prominent mammary infection in lactating cows results in high-titre shedding of infectious virus in milk, mastitis, and production losses. Animal movements contribute to subsequent cattle-associated spread, and occupational infections have occurred among dairy workers. The natural portal of entry, the relative contributions of milking-associated, respiratory, and environmental transmission, and whether cattle constitute a long-term reservoir remain unresolved. IDV is maintained in cattle populations through respiratory transmission, including direct-contact and short-range airborne spread. It diversifies during enzootic circulation and may contribute to disease within the polymicrobial setting of BRDC. Human exposure signals have been reported, but their clinical significance remains uncertain. This summary reflects evidence on human exposure, infection, and transmission available through 7 July 2026; dashed boxes indicate unresolved questions.

The clearest difference lies in the principal site of replication and predominant shedding matrix. In lactating dairy cows, H5N1 replication is especially prominent in mammary epithelium and produces high virus titers in milk (Baker et al., 2025). Bulk-tank milk is a scalable matrix for herd-level screening, but it contains only milk contributed to the tank and excludes withheld milk. It therefore provides no information on non-lactating cattle, and infection may be missed very early after introduction into a herd or when within-herd prevalence is low. IDV replicates in the respiratory tract and spreads by direct contact and short-range aerosols (Salem et al., 2019). Nasal or nasopharyngeal swabs are better suited to detecting active respiratory infection and, when collected longitudinally, to characterizing herd-level circulation, whereas lower-airway samples can support targeted BRDC investigations.

Genomic surveillance addresses distinct questions at different timescales. After H5N1 spillover, surveillance tracks outbreak expansion and repeated introductions (Food and Agriculture Organization of the United Nations et al., 2026) and examines substitutions arising during cattle-associated evolution (Dholakia et al., 2026). During enzootic IDV circulation, whole-genome analysis characterizes diversification and geographic spread (Limaye et al., 2024), cocirculating lineages and reassortment (Collin et al., 2015), and regional persistence (Li H. et al., 2026). Sequence change alone, however, is not evidence of increased zoonotic potential.

H5N1 and IDV detections carry different epidemiological implications. H5N1 detection in cattle or bovine milk should prompt rapid confirmation, whole-genome sequencing of representative positive samples, reconstruction of herd-to-herd transmission pathways and animal-movement networks, and assessment of epidemiologically linked animals and exposed workers. Emerging substitutions merit phenotypic testing when their frequency or context raises concern. IDV detection, by contrast, must be interpreted in light of assay-specific cycle threshold (Ct) values, sampling time relative to disease onset, transport history, lesions, and coinfecting BRDC pathogens. In most settings, an IDV-positive result should inform longitudinal investigation of respiratory disease rather than trigger HPAI-like emergency control measures.

## 5 Surveillance and control implications

Sampling is most informative when it targets the principal site of replication for each virus (**Figure 2**). In the United States, the National Milk Testing Strategy combines processing-plant milk-silo monitoring with state-specific surveillance, case investigation, and movement-associated testing under current federal guidance (United States Department of Agriculture, Animal and Plant Health Inspection Service, 2026). A positive bulk-tank test result or a cluster of cows with an abrupt decline in milk yield, abnormal milk, or unexplained mastitis warrants cow-level follow-up sampling, confirmatory RT-qPCR, review of cattle movements, and sequencing of viral genomes from representative positive samples. Because some cows shed virus in milk without overt signs, clinical surveillance alone is insufficient (Campbell et al., 2026).

**Figure 2:**
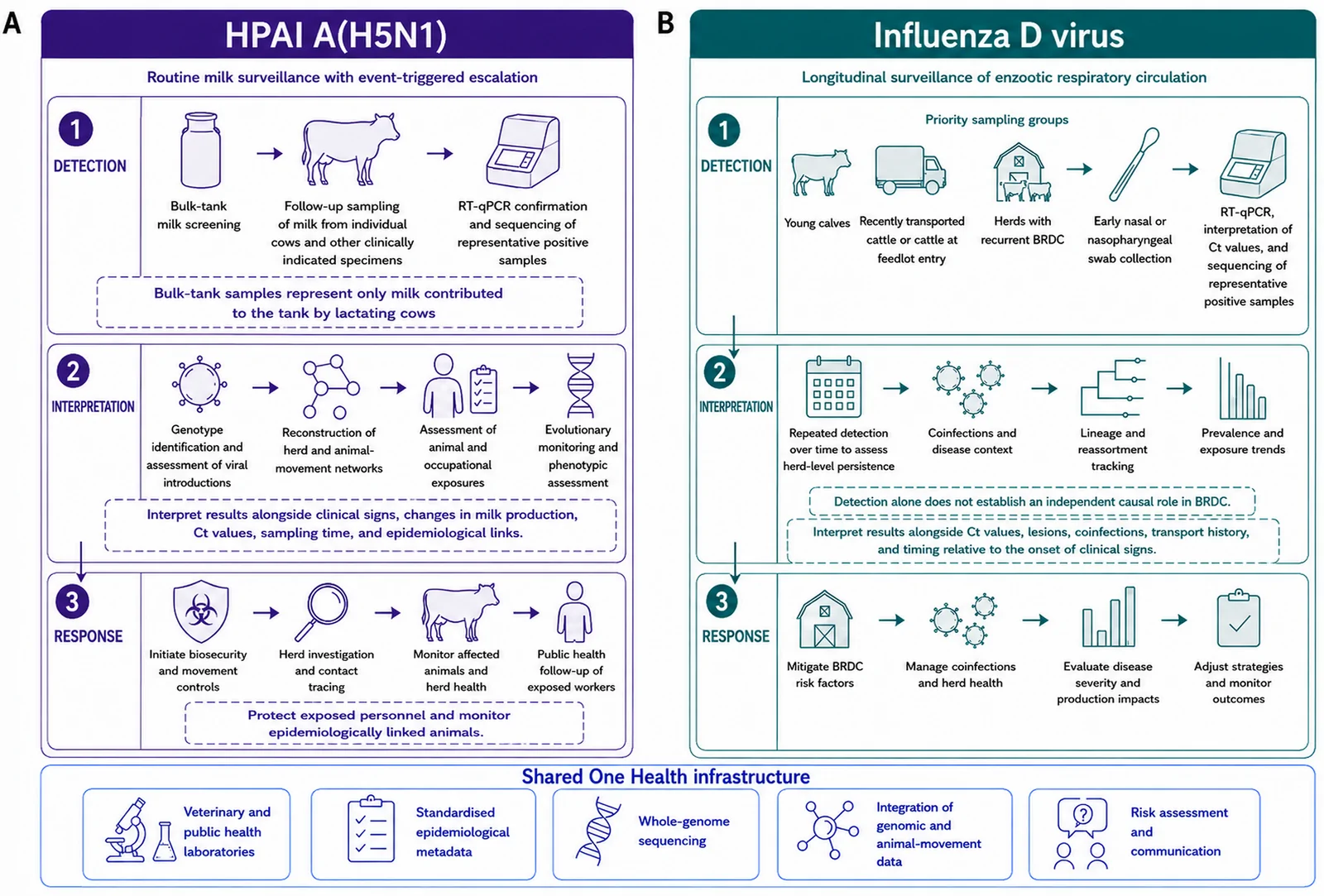
Proposed surveillance frameworks for HPAI A(H5N1) **(A)** and influenza D virus (IDV) **(B)** in cattle. The diagram synthesizes the evidence reviewed here and is not a regulatory algorithm. **(A)** links routine milk screening to cow-level follow-up and confirmatory testing after a positive screening result or a signal of epidemiological concern; bulk-tank samples contain only milk contributed to the tank by lactating cows. **(B)** combines repeated respiratory sampling with interpretation based on sampling time relative to clinical onset, lesions, coinfections, transport history, and assay context; detection alone does not establish that IDV independently causes BRDC. Both frameworks require laboratory capacity, genomic surveillance, movement data, and risk communication. For IDV, “early” sampling refers to collection soon after transport or feedlot entry or near the onset of respiratory signs. Cycle threshold (Ct) values are assay-dependent and should not be compared directly across assays or laboratories.

Environmental sampling is most useful during outbreak investigations. Milking-parlour air, wastewater, and frequently contacted surfaces can reveal contamination and potential exposure points, but environmental detection neither proves a transmission route nor replaces animal-level testing (Campbell et al., 2026). A kinetic model estimated a median interval of approximately 7.8 days during which milk-sample Ct values remained below a model-inferred threshold associated with the likely presence of infectious virus (Eales et al., 2026). This estimate depends on pooled data, limited longitudinal observations, and assumptions linking Ct values to infectivity; it should not be treated as a fixed infectious period or regulatory clearance interval.

Longitudinal respiratory surveillance is more informative for IDV. Priority groups may include young calves, recently transported cattle, cattle at feedlot entry, and animals from herds with recurrent BRDC. Sampling soon after transport or near respiratory-sign onset can address different objectives. Assay-dependent Ct values require interpretation with specimen quality, clinical findings, lesions, coinfections, transport history, and epidemiological context and are not directly comparable across assays or laboratories. Selected lower-airway samples can help relate viral replication to lesions and bacterial coinfection. Paired serology supports recent exposure but cannot identify the replication site or transmission direction. Whole-genome sequencing of representative positives supports lineage and reassortment analysis. IDV surveillance is program-specific; the HPAI notification requirements applied to H5N1 in cattle do not apply to IDV (World Organisation for Animal Health, 2025).

The two surveillance strategies share core infrastructure: validated diagnostics, whole-genome sequencing, and standardized metadata on host characteristics, specimen type, sampling date, clinical status, sampling location, movement history, exposure history, and co-pathogens. For H5N1, WHO guidance recommends monitoring exposed people and reporting suspected or confirmed human infections after relevant animal detections (World Health Organization, 2025). Guidance from the WOAH-FAO Network of Expertise on Animal Influenza (OFFLU) addresses cattle surveillance, biosecurity, risk mitigation, and cross-sector data sharing (OFFLU, 2025). Integrating genomic and movement data can help reconstruct spread through production networks.

Following H5N1 detection, control measures should reflect local epidemiology and applicable regulations. Current OFFLU guidance recommends managing cattle movements, separating infected animals, handling waste milk and wastewater safely, cleaning milking areas, and protecting personnel (OFFLU, 2025). WOAH requires Members to notify occurrences of HPAI in cattle through the World Animal Health Information System (WAHIS) and recommends that viral sequences be shared publicly (World Organisation for Animal Health, 2025). Vaccination may eventually complement surveillance and biosecurity, but evidence from cattle remains preliminary. A dual-route H5 vaccine induced systemic and mucosal responses in calves, but its protective efficacy against mammary infection, milk shedding, and cattle-to-cattle transmission has not been evaluated in a bovine challenge study (Wiggins et al., 2026).

Current evidence does not support applying an HPAI-like emergency response solely on the basis of routine IDV detection. Improving disease attribution will require prospective BRDC cohort studies, standardized coinfection models, and whole-genome sequencing of representative positive samples. Future studies should distinguish exposure from productive infection and productive infection from attributable disease. Such evidence would support proportionate interventions focused on BRDC risk factors and herd health rather than emergency population-level control.

## 6 Discussion

Although both viruses infect cattle, their assessment requires different risk frameworks. Epidemiological consequences depend on the principal replication site and its relationship to production. In H5N1-infected dairy cows, mammary infection links viral shedding to milk production and milking practices. High-titer shedding in milk creates a direct exposure pathway; animal movements facilitate dissemination, while occupational contact creates opportunities for human exposure. IDV lacks this production-linked route; instead, it is maintained by respiratory transmission and interacts with other pathogens within the polymicrobial setting of BRDC. The most informative samples, genomic priorities, and response thresholds therefore differ.

The principal uncertainties differ between the two viruses. For H5N1, the natural portal of entry, the relative contributions of milking-associated, respiratory, and environmental transmission, and whether cattle constitute a long-term reservoir remain unresolved. For IDV, respiratory replication and cattle-to-cattle transmission are established, but its independent contribution to naturally occurring BRDC remains uncertain, as does the clinical significance of human exposure signals. Resolving these questions will require studies that integrate viral kinetics with tissue lesions, co-pathogen dynamics, animal-movement histories, clinical outcomes, and transmission phenotypes.

The supporting evidence is asymmetric in scope and design. H5N1 evidence derives mainly from intensively investigated outbreaks in lactating United States dairy cattle and a few experiments; extrapolation to non-lactating cattle, beef cattle, calves, and other production systems remains uncertain. IDV studies span more regions and years but often use cross-sectional PCR or serology, heterogeneous sampling, and incomplete genomic data. These differences preclude simple comparisons of prevalence, transmission efficiency, pathogenicity, or zoonotic risk. Preparedness should therefore reflect virus-specific replication sites and production systems rather than host species alone.
